# Comparison between Bipolar Hemiarthroplasty and Total Hip Arthroplasty for Unstable Intertrochanteric Fractures in Elderly Osteoporotic Patients

**DOI:** 10.1371/journal.pone.0039531

**Published:** 2012-06-22

**Authors:** Lihong Fan, Xiaoqian Dang, Kunzheng Wang

**Affiliations:** Department of Orthopaedic Surgery, Second Affiliated Hospital, Medical School of Xi’an Jiaotong University, Xi’an, Shaanxi Province, People's Republic of China; University of Texas Southwestern Medical Center, United States of America

## Abstract

The present study was conducted to compare bipolar hemiarthroplasty (BA) with total hip arthroplasty (THA) in treatment of unstable intertrochanteric fractures in elderly osteoporotic patients. The THA group included 14 males and 26 females with a mean age of 73.4 years, and the BA group included 27 males and 45 females with a mean age of 76.5 years. Significant difference existed between the two groups in operation time, blood loss, transfusion volume and cost of hospitalization, while no remarkable difference was identified in hospitalization period, general complications, joint function, pain, rate of revision and mortality. No dislocation was observed in BA group while 3 occurred in THA group. The results indicated that for unstable intertrochanteric fractures in elderly osteoporotic patients, BA seems to be a better or more reasonable choice compared with THA for the reason of less blood loss, shorter operation time, lower cost and no dislocation.

## Introduction

Intertrochanteric fractures of the femur often occur in elderly people. Their incidence has increased due to the increased life expectancy and osteoporosis [1]. Rigid internal fixation and early mobilization are the key points of the treatment. Stable intertrochanteric fractures can be easily treated by osteosynthesis with predictable good results [2], [3], whereas the management of unstable intertrochantric fractures is challenging because of poor bone quality, osteoporosis and other underlying diseases [4], [5]. Although there are some fixation methods such as fixed nail plate, sliding hip screw and intramedullary interlocking devices, no one guarantees absolute fracture stability and complete bone union in elderly patients [6]–[8]. Osteoporosis and instability are two of the most important factors leading to unsatisfactory results of treatment [9], [10], and in the elderly the coexistence of unstable, comminuted fractures with osteoporosis worsens the prognosis [11].

Due to high failure rate and complications associated with internal fixation, prosthetic replacement has been recommended by some authors as primary treatment for unstable intertrochanteric fractures [12]–[14]. There have been various reports of successful outcomes after the use of hemiarthroplasty and total hip arthroplasty (THA) [15]–[17]. Stappaerts et al., in a prospective randomized study, concluded that primary cemented endoprosthesis brought better results than compression hip screw in unstable intertrochanteric fractures in elderly osteoporotic patients who were eligible for early mobilization [16]. Parvjeet found in his study that the patients treated with bipolar prosthesis had earlier rehabilitation than those treated with internal fixation, which decreased the overall morbidity, and he therefore concluded that bipolar prosthesis might be favored in old-aged patients even though there was no major difference in the choice of either implant [17]. After hip arthroplasty, patients can bear weight immediately and are encouraged to move and exercise the involved limbs, and thus reduce the period of bed rest and the rate of complications.

Bipolar hemiarthroplasty (BA) is a less complicated and more expensive surgery compared with THA [18]. However, no study is available that compares the effects and outcomes of the two treatments in intertrochanteric fractures. In order to get a clearer picture about the difference in their performance, we conducted the present study to compare the clinical effects of BA and THA in elderly osteoporotic patients with unstable intertrochanteric fractures, in terms of operation time, blood loss and transfusion, duration and cost of hospitalization, hip joint function, pain relief, general complications, and the rate of dislocation, revision and mortality.

## Methods

All the patients with intertrochanteric fractures admitted to the hospital between March 2003 and September 2009 were evaluated. This study was a retrospective study of prospectively collected data. We used Singh's classification of the trabecular bone structure in the proximal femur as a measure of osteoporosis based on the anteroposterior (AP) radiograph of the contralateral hip. The inclusion criteria were: unstable intertrochanteric fractures (three or more part intertrochanteric fractures with a loss of posteromedial cortical buttress and reverse obliquity fractures), age over 70 years, severe osteoporosis (Singh index ≤3), no contraindication to anaesthesia, and pre-injury independent walking with or without aids. The exclusion criteria were: suspected pathological fracture, significant senile dementia, and osteoarthritis or rheumatoid arthritis in the fractured hip.

All patients were treated operatively with prosthetic replacement by the same surgery team. Follow-up evaluations were performed at 6 weeks, 3, 6, 9 and 12 months, and every year thereafter. THA was performed on the patients admitted in the hospital before December 31, 2005, but it was substituted by BA later in 2006 after the occurrence of three dislocations and for the sake of reducing dislocation rate and cost. The data of the patients’ hospital courses were collected by chart abstracting.

All the patients underwent surgery with a standard posterior approach in lateral position under spinal or general anesthesia within 48 hours of admission. The fibres of the gluteus maximus were split and then the gluteus medius was retracted to expose the short external rotator muscles of the hip. These were divided close to their insertion and an inverted T shaped incision was made on the joint capsule. To avoid a further displacement of the fragments, with the limb maintained in traction by the surgical assistant, osteotomy of the femoral neck was performed prior to the dislocation of the hip joint. The femoral head was removed. The fragments of the greater trochanter were repositioned and temporarily fixed by using one or two bone forceps. The femoral canal was carefully detected with a long spoon and then was prepared by graduated reaming using rasps. Anteversion-retroversion of the prosthesis was determined using the lesser trochanter as a guide after the lesser trochanter was temporarily reduced. The height of the prosthesis was determined by temporarily fixing the greater trochanter in its anatomical position. In severely comminuted fractures, it was difficult to determine the prosthesis height properly only by anatomical landmarks of trochanters. Trial stem was used to decide the appropriate length of the extramedullary portion of the femoral component. The trial stem was assembled with a trial cup, and reduction test was performed to determine the exact length of the prosthesis that would achieve equal limb length.

The second-generation cementing technique was utilized. Thorough bony bed cleaning was performed by high speed pulsatile lavage. An intramedullary cement plug was placed, and the cement in a doughy state was delivered using a cement gun in a retrograde fashion. The Centrament® stem (Aesculap, Tuttlingen, Germany), with a length of 180 to 220 mm, was inserted inside the femoral canal and positioned at an anteversion angle of 15°. No obvious cardiopulmonary complications were observed. The fractured greater trochanter was attached to the prosthesis with two to four 16-gauge stainless steel wires. Isolated displaced fragments of the lesser trochanter were not reduced and fixed. In the THA group, the acetabulum was prepared and a cementless cobalt-chromium cup (Aesculap) with a UHMWPE liner inside was implanted, and then a 28-mm metal head was attached to the femoral stem. The optimal socket position was 40° to 45° of abduction and 10° to 25° of anteversion. Higher range of anteversion was preferred in order to reduce the risk of posterior instability. In the BA group, the acetabulum was not replaced, and a bipolar cup (Aesculap) was implanted instead. After the femoral head was removed, the diameter of the head was measured to determine the approximate size of the outer head of the chosen prosthesis. Range of motion and stability were checked after reduction. The capsule was repaired followed by reattachment of the short external rotators to the femur. Routine closure was performed and vacuum drainage was placed in both groups.

All patients underwent a routine postoperative physiotherapy protocol. The vacuum drains remained in place for 48 hours and were then removed. A pillow between the thighs was used for the first 2 weeks to prevent excessive adduction when the patients lied on the unoperated side. The patients, according to their conditions, were made to sit or stand with support from the first to the third postoperative day, and ambulated with support within the third to the fifth postoperative day. The rehabilitation progressed based on the toleration of the patients. Prophylaxis against deep venous thrombosis using Low-molecular-weight heparin (Lovenox 40 mg) was started 12 hours prior to the operation and continued for 35 days postoperatively.

At the final follow-up, the functional outcome was evaluated using Harris Hip Score (HHS) and the degree of pain was measured by visual analogue scale (VAS). Anteroposterior radiographs of the hip were taken at each follow-up for the evidence of subsidence of the stem, migration of acetabular component, erosion of acetabulum, and heterotopic ossification. The operation and medical records were reviewed to get the information of operation time, blood loss, blood transfusion volume, duration and cost of hospitalization.

All procedures used for this study were reviewed and approved by the Institutional Review Board of the Second Affiliated Hospital of Xi’an Jiaotong University, China. Written informed consent was obtained from participants after adequate explanation of the procedures of the study. Approval by the Institutional Review Board was documented.

The results were compared between the two groups for statistical significance either by a Student’s t-test or a Mann-Whitney U test. Dichotomous variables, such as rates of revision and displacement, were analysed using a *chi*-squared test or Fisher’s exact test. The p-value less than 0.05 was considered statistically significant.

## Results

Between March 1, 2003 and September 30, 2009, a total number of 156 patients were admitted with a diagnosis of intertrochanteric fractures. Among the patients, 20 had severe hip arthritis, 16 had significant dementia, and 8 were excluded for pathological fractures. As a result, 112 patients that met the selection criteria were included. For these patients, 40 received THA and 72 underwent BA. The BA group had a mean follow-up period of 39.7 months (24 to 62 months) and the THA group had 48.8 months (32 to 75 months). The difference in the follow-up period between the two groups was statistically significant (p<0.01).

The demograghic characteristics of the 112 patients are summarized in Table 1. The THA group included 14 males and 26 females with a mean age of 73.4 years (range 70–80 years), and the BA group included 27 males and 45 females with a mean age of 76.5 years (range 71–85 years). Most patients had comorbidities that could adversely affect the functional outcomes, such as cardiovaslular problems, diabetes mellitus, pulmonary diseases and other associated diseases, but there was no significant difference in the number of comorbidities between the two groups. The data including age, sex, BMI, fracture type and Singh index of patients in the two groups also showed no significant difference.

**Table 1:** Patient demographics.

	THA	BA
men:woman	14:26	27:45
Age (year)	73.4±5.8	76.5±8.3
BMI	22.3±4.7	25.1±5.2
Fracture type(IIIa)	12	23
Fracture type(IIIb)	22	33
Fracture type(IV)	6	16
Singh index	2.9±0.7	2.8±0.5
Follow-up (mon)	48.8±15.2	39.7±13.3
No. of Comorbid conditions	0.8±0.4	0.9±0.3

THA: total hip arthroplasty; BA: Bipolar hemiarthroplasty: Fracture type: Evans/Jensen classification system.

The detailed surgery information of the patients is given in Table 2. The mean operation time in the THA group was 74.5 min, much longer than 53.4 min in the BA group. The average blood loss of the THA patients was 475.3 ml, two times of the blood loss of 252.8 ml of the BA patients, and the average blood transfusion volume in the THA group was even more than two times that in the other group. The differences between the two groups in operation time, blood loss and transfusion volume were significant (P<0.05). It can be also seen that although the patients stayed in hospital for similar length of duration, the costs of hospitalization in the two groups were remarkably different. The THA patients spent a lot more than the BA patients did.

**Table 2:** Operative records of patients.

	THA	BA	p-value
Operation time (minute)	74.5±15.2	53.4±12.5	<0.001
Blood loss (ml)	475.3±122.4	252.8±82.6	<0.001
Blood transfusion volume (unit)	3.5±1.2	1.5±0.8	<0.001
Hospitalization duration (day)	18.7±4.9	18.2±5.1	0.615
Hospitalization cost (thousand Yuan)	47.2±13.5	35.4±11.2	<0.001

THA: total hip arthroplasty; BA: Bipolar hemiarthroplasty.

The mean HHS was 76.8 in the THA group and 74.6 in the BA group, and the mean VAS was 1.6 in the former and 1.8 in the latter group. Nine patients in the THA group (22.5%) and 16 patients in the BA group (22.2%) had two or more general complications. No significant difference was found between the two groups in HHS, VAS and general complications. For local complications, three dislocations occurred in the THA group while none was observed in the BA group, and this difference was statistically significant (p<0.05). One dislocation was treated by revision while the other two were closely reduced, and no recurrence was observed in the follow-up. During follow-up, two patients (4.4%) in the THA group and three patients (4.2%) in the BA group underwent a revision operation, eleven patients (27.5%) in the THA group and nineteen patients (26.3%) in the BA group died. The difference between the two groups in revision rate and mortality rate was not significant (p>0.05) (Table 3).

**Table 3:** Outcome at the end of follow-up.

	THA	BA	p-value
Mean HHS	76.8±17.1	74.6±15.3	0.486
Mean VAS	1.6±0.6	1.8±0.5	0.062
Complications (%)	9(22.5)	16(22.2)	0.973
Dislocation (%)	3(7.5)	0(0)	0.018
Revision operation (%)	2(5.0)	3(4.2)	0.838
Mortality (%)	11(27.5)	19(26.3)	0.899

THA: total hip arthroplasty; BA: Bipolar hemiarthroplasty; The total modified Harris Hip score (HHS) was converted to a maximum of 100 points; HHS, VAS: students t-test; mortality: chi-squared test; revision operation, dislocation: Fisher’s exact test.

**Figure 1:**
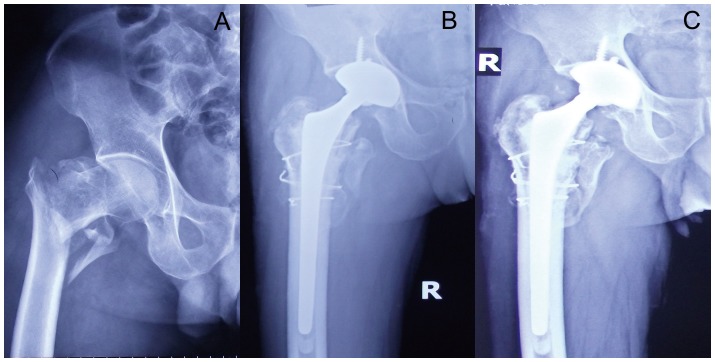
AP hip radiographs of a 76-year-old man with severe osteoporosis and unstable intertrochanteric fracture treated with cement THA using a long stem. A: Preoperative; B: At 6 months postoperatively, the fractured fragment has healed; C: Four years postoperative, the prosthesis was well fixed and his Harris hip score was excellent.

**Figure 2:**
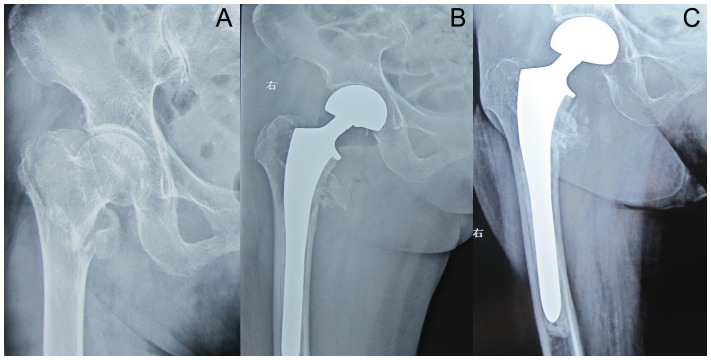
AP hip radiographs of a 78-year-old woman with severe osteoporosis and unstable intertrochanteric fracture treated with cement bipolar hemiarthroplasty using a long stem. A: Preoperative; B: At 6 months postoperatively, the fractured fragment has healed; C: radiographs obtained three years postoperatively, Femoral component was stable with no protrusion of cup.

Radiography at the last available follow-up showed that all greater trochanter fractures had healed (Figure 1 and 2). The cerclage wire used for the greater trochanter was found broken in two patients in each group. Only one patient in the BA group developed osteoarthritis of the acetabulum and required a revision to THA because of pain in the groin. The evidence of loosening of femoral component was seen in two BA patients and one THA patient, and revision was performed. There was no sign of heterotopic bone formation in any of the patients.

## Discussion

The incidence of all hip fractures is approximately 80 per 100,000 persons and is expected to double over the next fifty years as the population ages [19]. According to the criteria of the modified Evans-Jensen classification, the two-part fractures are considered stable fractures and the rest of the fractures are unstable. About 35%–40% of all intertrochanteric hip fractures are unstable three- and/or four-part configurations with displacement of the posterior-medial cortex [20]. The failure rate of unstable intertrochanteric fractures with osteoporosis has been reported to be between 4% and 16.5% [21], [22].

The dynamic hip screw and proximal femoral nail have been commonly used for internal fixation of intertrochanteric fractures. Elderly osteoporotic patients with unstable intertrochanteric fractures usually have a high prevalence of unsatisfactory outcome, with shortening and external rotation deformity of the limb following the treatment with a sliding screw. The failure rate of the dynamic hip screw in unstable fractures is up to 14% [23], [24]. The use of intramedullary nail is associated with complications such as screw migration, femoral shaft fracture and implant failure. The failure rate of the proximal femoral nail is between 7.1% and 12.5% [8], [25].

The incidence of general complications such as pulmonary embolism, deep venous thrombosis and pneumonia ranges from 22% to 50% when internal fixation was adopted [26], [27]. Complications are also related to postsurgery rehabilitation, such as duration of bed rest and starting time of weight bearing [28]. Comminution, osteoporosis and instability often preclude the early resumption of full weight-bearing and worsen the prognosis. Mortality rate in hospital ranges from 0.03 to 10.5% [29], while one-year mortality reaches 22% [30].

Primary THA and hemiarthroplasty have been used to treat unstable intertrochanteric fractures in an effort to mobilize the patients more rapidly and avoid complications of hip screw migration [31]–[34], and they are found to have many merits against other fixation techniques. In a recent study, Faldini et al. reports the use of hemiarthroplasty and THA in 54 patients [15] and the finding that hip replacement permits a more rapid recovery with immediate weight-bearing and facilitates nursing care better than other fixation techniques. Sidhu et al also proved in their study on 53 patients that THA may be a valid treatment in mentally healthy elderly patients with intertrochanteric hip fractures [12]. Hemiarthroplasty and THA, as two possible treatment options for unstable intertrochanteric fractures, may offer the potential for quick recovery with little risk of mechanical failure, avoid the risks often associated with internal fixation, and enable patients to maintain a good level of function immediately after surgery. Meanwhile, they do not result in nonunion or malunion of the fracture site or complications associated with avascular necrosis of the femoral head.

We have noticed that in treating femoral neck fractures, BA, compared with THA, has the advantages of less complexity, shorter operation time and a lower probability of dislocation. However, it may introduce concerns regarding groin and thigh pain due to acetabular erosion which reduces long-term survival and increases the likelihood of a second operation. For deciding whether BA or THA is a better choice for patients with intertrochanteric fractures, four factors should be taken into consideration: operation wound, surgery outcome, complications associated with procedures such as dislocation and acetabular erosion, and operation cost.

In our study, no significant difference has been identified between the group of patients treated by THA the patients treated by BA in surgery outcome, revision rate, mortality rate and general complications. However, the operation time of THA is evidently longer, and the blood loss and blood transfusion volume in the THA patients are significantly higher. The estimated cost of the components in THA is higher than that in BA, and the additional acetabular component in THA requires extra expense. In addition, the increased blood transfusion and longer operation time also increase the hospital cost. In resource-poor countries like China, cost is one of the major factors in patients’ selection of treatments.

Haentjens [35] et al reviewed the literature and summarized the reports regarding prosthetic replacement for the treatment of intertrochanteric fractures and their complications. They concluded that elderly patients with severe osteoporosis may benefit from prosthetic replacement for comminuted intertrochanteric fractures and non-unions. Few serious orthopaedic complications are associated with the procedure and most patients have good pain relief. The major concern post THA is dislocation, which increases the rate of pulmonary complications and bed sores [36]. The reported rate of dislocation in patients with intertrochanteric fractures after THA is up to 44.5% [34]. Despite that larger heads may give better stability, a larger bearing alone cannot prevent dislocation [37]. The standard metal ball with a diameter of 28 mm and the polyethylene cup have been in use since early 1960s and have been the most used till now. It is also the least expensive bearing. In our series, no dislocation occurred in the BA patients during follow-up, while three of the THA patients suffered dislocations. One patient with mal-positioning of the stem and acetabular component underwent revision after the failure of closed reduction. The other two patients received closed reduction under general anesthesia, and were protected with an abduction brace for two months. No recurrence was then observed. Bracing was not routinely practiced after THA surgery but only after closed reduction of dislocation, because it would immobilize the hip joints, which was poorly tolerated by elderly patients, and was a considerable expense.

The main concern with BA is the possibility of occurrence of protrusion acetabuli and groin pain from the acetabular erosion [38], [39]. Cartilage damage and erosion of the acetabular surface are highly correlated with groin pain [40]. Compromised articular cartilage in the hips of normal elderly patients puts them at a greater risk. In our study, although VAS was slightly higher in the BA group, there was no statistically significant difference between the two groups. Patients with intertrochanteric fractures caused by osteoporosis often have coexisting medical problems and limited life expectancy, which means that the daily demands placed on prostheses are low [41]. It is also found in our study that more than 25% of the patients (19 out of 72) expired within 5 years post surgery, long before severe protrusion would occur. Obvious acetabular erosion was observed in only one BA patient, who underwent THA as a revision since the pain in the groin was not relieved by oral administration of NSAIDs.

This is a retrospective study, which bears some limitations. The number of cases included may not be large enough, and the follow-up period is relatively short. A further prospective, randomized study is necessary to compare the arthroplasty procedures in more cases with longer follow-up.

The present study reveals that the functional results and pain relief in patients undergoing THA and BA are similar, and there is no evident difference in hospitalization period, general complications, and rate of revision and mortality during follow-up. Both THA and BA are reliable treatment methods for unstable intertrochanteric fractures in elderly osteoporotic patients. However, the higher intra-operative blood loss, longer duration of surgery, higher incidence of dislocations, and greater costs of THA suggest that BA might be a better or more reasonable choice.
